# Virtues of Medical Men

**Published:** 1855-09

**Authors:** 


### VIRTUES OF MEDICAL MEN.

The valedictory of Dr. Morse Stewart to the Graduating Class of the Medical Department of the University of Michigan, is truly an able document. abounding in truths most forcibly and happily expressed. We would that we could publish it entire. The following however is so excellent and so much in accordance with our views that we cannot refrain from extracting it. We have ever regarded the profession as a sacred mission, and quite too much so to make it a *cent per centum* consideration. By common consent it is regarded in this light by the true profession, for so soon as a desire for greater accumulation obtains, then the profession is abandoned for another and a more profitable field of operation.

We must confess that when we find a medical man looking to the proceeds of his professional labors as the bright road to wealth and opulence, we entertain serious fears that his attainments will be neglected, and that if he manifests a desire for improvement, it is only because he can make it profitable to himself individually. Such aspirations are too sordid and quite too selfish for the sacred vocation which he has unwisely chosen, for if he expects wealth, without compromising the dignity of the profession, he has deceived himself.

A physician in active practice is in a poor condition to attend to his finances. He must neglect one or the other, more or less.— And yet community is cold enough in some instances to condemn a medical man as "worse than an infidel" if he is not always in pursuit of his gains, with his pocket crammed to repletion with bills and accounts. Suppose that he is on the wing, in active pursuit of the "almighty dollar" and at the same time a bed-ridden patient is neglected because of it, what would he think if he knew the cause of his medical attendants temporary abandonment of him? He would be condemned as an unfeeling misanthrope, and bitter maledictions would be showered upon him. Community are wrong when they indulge in such unjust reflections, and in order to obviate it let them call upon their physician, contribute to his wants by settling their bills soon after the performance of the services or if not, they should not indulge in reflections, if the physician is pressed with his labors so closely as not to be able to call upon them. Some men more fortunate in their organization than others can, without compunction or scruple, bluntly dun their patrons for the amounts due them, and there are others too who have the tact to be able to do so without even giving offence, but it is not so with others. There is something revolting to good taste on the part of a physician to become clamorous for his fees, when if he indulges in a little retrospect he will find that, to put himself in possession of these golden gains, he has been to the bedside of suffering, with all its sorrowing and agonizing inflictions, he has heard the appealing moan, he has seen the tear-drop fall from the eye of sorrow-stricken relatives and friends, he has heard the heart rending appeals for relief, and has witnessed the last moments of his patient and friend and the sorrow and mourning of relatives for the loss of one dearly loved—did he, under all these circumstances, made sacred by untold sufferings, afflictions, and misery, stand there unmoved by the scene transpiring before him, looking the very personification of sordid gain, rejoicing in the oppurtunity by which his greed may be satisfied? Surely if his face did not show that such were his thoughts, the bill for his fee, put into the hands of the afflicted family before the tracks of the bier which conveyed the remains of their relative to the silent tomb had yet been erased from the pathway to the sepulchre, would be at least presumptive proof that when he stood there no sorrow was in his heart,—no sympathy in his bosom, and no humanity in his nature. But to the extract from the published address in the Peninsular Journal, Ann Arbor, Michigan:

"Be not led astray by this common mistake. The physician and surgeon is not by any necessity of his calling, hard hearted and indifferent to the suffering he witnesses, since to witness pain, or even to occasion it, for the purpose of curing or relieving the suffering patient, *fosters* the tender sensibilities of the heart, and refines our human sympathies. It *may* be otherwise, and, no doubt, often is; for, if avarice, or ambition be the motive which prompts to the choice and practice of medicine, the love of self is exalted above the love of our neighbor; the claims of humanity are wholly lost sight of, and the sympathies of the man are dried up before the scorching, withering influence of the dominant purpose of the soul.

Cultivate, then, this noble virtue. Start upon your career of practice, deeply imbued with the benevolent character of your profession, and with the determination to maintain its character, and that its honor shall ever remain unsullied by any selfishness on your part. Engage in the practice of medicine, not as a means to enrich yourself, nor even as a *mere* livelihood, but with the high purpose of devoting your life to usefulness. Let it be your aim to do good to others as you have opportunity, and an approving conscience will sustain and comfort you—a better recompense, far, than money, for the labor and anxiety you must endure. So shall ye be classed among "physicians of that nobler kind," who

"Join with care and skill,
A temperate judgment, a devoted will;
Men who suppress their feelings, but who feel
The painful symptoms they delight to heal;
Patient in all their trials, they sustain
The starts of passion, the reproach of pain;
With hearts affected, but with looks serene,
Intent they watch through all the solemn scene,
Glad if a hope should rise from nature's strife,
To aid their skill and save the lingering life;
But this must virtue's generous effort be,
And spring from nobler motives than a fee."

But if from sordid motives you have chosen this profession and determined to practice it, you may succeed in your purpose; yet will not the happiness, which the higher incentive brings, attend you—and though you may receive your reward, yet if the vicissitudes of life, should suddenly dissipate your accumulated wealth, or blast your ambitious hopes forever, from whence could you look for consolation?"

